# Phylogenetic resolution of the fly superfamily Ephydroidea–Molecular systematics of the enigmatic and diverse relatives of Drosophilidae

**DOI:** 10.1371/journal.pone.0274292

**Published:** 2022-10-05

**Authors:** Isaac S. Winkler, Ashley H. Kirk-Spriggs, Keith M. Bayless, John Soghigian, Rudolf Meier, Thomas Pape, David K. Yeates, A. Bernardo Carvalho, Robert S. Copeland, Brian M. Wiegmann

**Affiliations:** 1 Department of Biology, Cornell College, Mount Vernon, Iowa, United States of America; 2 African Natural History Research Trust, Leominster, Herefordshire, United Kingdom; 3 Australian National Insect Collection, CSIRO National Research Collection, Australia (NRCA), Acton, Canberra, ACT, Australia; 4 Department of Comparative Biology and Experimental Medicine, Faculty of Veterinary Medicine, University of Calgary, Calgary, Alberta, Canada; 5 Department of Entomology & Plant Pathology, North Carolina State University, Raleigh, North Carolina, United States of America; 6 Department of Biological Sciences, National University of Singapore, Singapore, Singapore; 7 Natural History Museum of Denmark, Copenhagen, Denmark; 8 Departamento de Genética, Instituto de Biologia, Universidade Federal do Rio de Janeiro, Rio de Janeiro, Brazil; 9 International Centre of Insect Physiology and Ecology (ICIPE), Nairobi, Kenya; Universita degli Studi di Roma La Sapienza, ITALY

## Abstract

The schizophoran superfamily Ephydroidea (Diptera: Cyclorrhapha) includes eight families, ranging from the well-known vinegar flies (Drosophilidae) and shore flies (Ephydridae), to several small, relatively unusual groups, the phylogenetic placement of which has been particularly challenging for systematists. An extraordinary diversity in life histories, feeding habits and morphology are a hallmark of fly biology, and the Ephydroidea are no exception. Extreme specialization can lead to “orphaned” taxa with no clear evidence for their phylogenetic position. To resolve relationships among a diverse sample of Ephydroidea, including the highly modified flies in the families Braulidae and Mormotomyiidae, we conducted phylogenomic sampling. Using exon capture from Anchored Hybrid Enrichment and transcriptomics to obtain 320 orthologous nuclear genes sampled for 32 species of Ephydroidea and 11 outgroups, we evaluate a new phylogenetic hypothesis for representatives of the superfamily. These data strongly support monophyly of Ephydroidea with Ephydridae as an early branching radiation and the placement of Mormotomyiidae as a family-level lineage sister to all remaining families. We confirm placement of Cryptochetidae as sister taxon to a large clade containing both Drosophilidae and Braulidae–the latter a family of honeybee ectoparasites. Our results reaffirm that sampling of both taxa and characters is critical in hyperdiverse clades and that these factors have a major influence on phylogenomic reconstruction of the history of the schizophoran fly radiation.

## Introduction

In the vast topology of the phylogenetic tree of life, we occasionally encounter clades so unlike any relatives that their placement remains uncertain. Many of these have specialized lifestyles that have probably contributed to the evolution of their modified or “unusual” morphologies. In particular, many lineages of parasites have been especially difficult to place phylogenetically using morphological characters. Classic examples abound in the insects [1], flowering plants [2], bacteria [3], fungi [4, 5] and other metazoans [6]. Although many of these “orphan” lineages have found a home in the era of molecular systematics [7–9], even large phylogenomic data sets are sometimes unable to resolve challenging phylogenetic questions surrounding these lineages [1]. That these puzzles persist has been variously attributed to rapid radiations, adaptations that obscure groundplan synapomorphies and unpredictable conflict among data types. Clearly, a general lack of corroborating evidence, along with potential problems of rate heterogeneity, model misspecification and the effects of uneven sampling can limit the resolving power of large molecular data sets [10, 11]. These data-specific issues are further exacerbated by uneven taxon sampling, that in these cases is both natural–due to imbalanced diversification and extinction through time; and operational–due to rarity and challenges of obtaining and studying taxa with restricted geographic ranges and specialized life histories.

**Fig 1:**
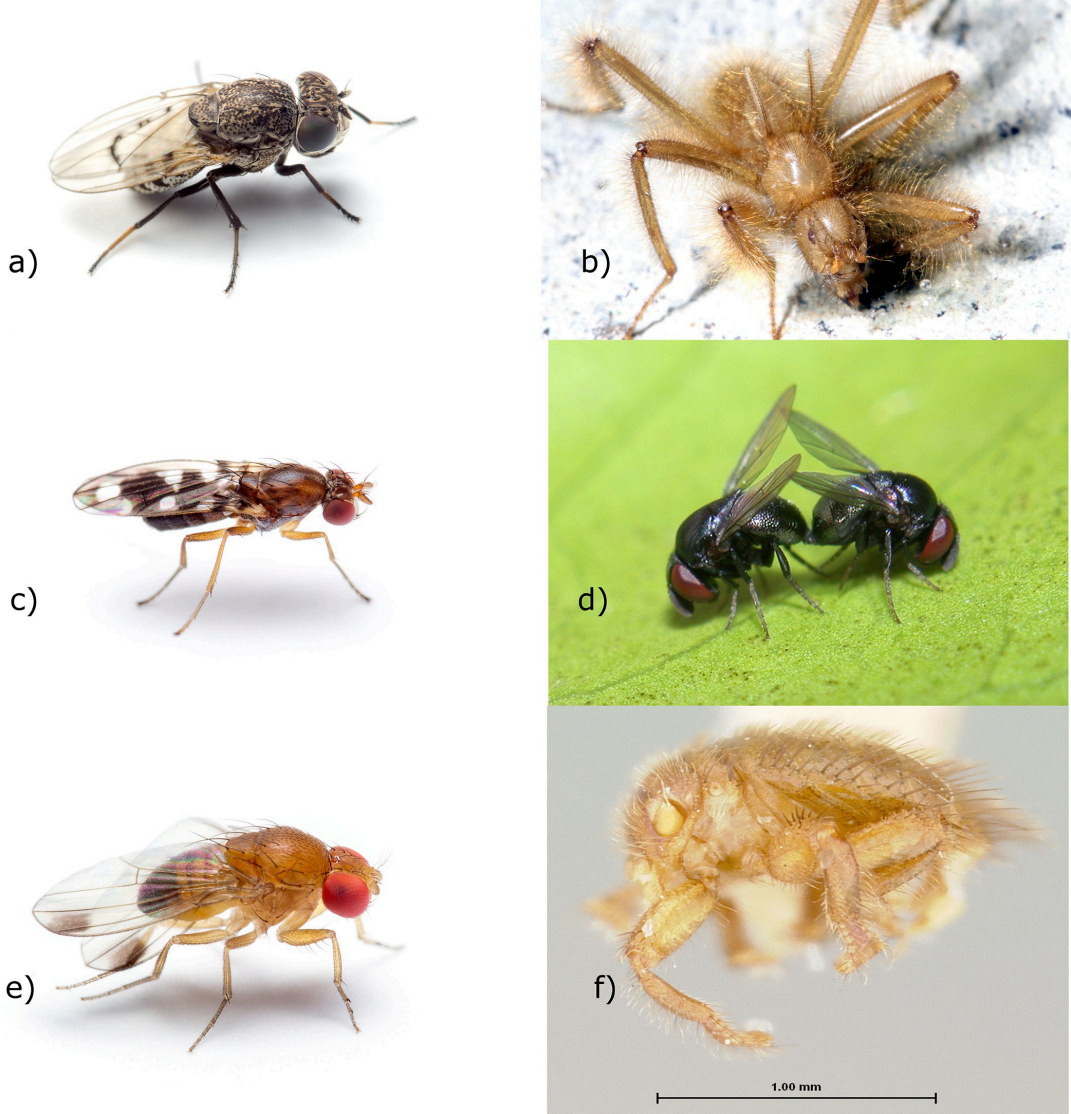
Representatives of the diverse adults of Ephydroidea: a) Ephydridae: *Paralimna punctipennis* (Wiedemann); b) Mormotomyiidae: *Mormotomyia hirsuta*; c) Diastatidae: *Diastata* sp.; d) Cryptochetidae: *Cryptochetum* sp.; e) Drosophilidae: *Drosophila suzukii*; f) Braulidae: *Braula coeca*. Photographs: Fig 1A, 1C, 1E: M. Bertone; Fig 1B: R.S. Copeland; Fig 1D: A. Wild; Fig 1F: K.M. Bayless.

Among the Diptera, there are few species as enigmatic as the wingless, solifuge-like “terrible hairy fly”, *Mormotomyia hirsuta* Austen [12] (Fig 1B). Formerly known only from a cave-like rock cleft in Kenya [13, 14], this species was rediscovered in 2010 [15, 16] at the same locality from which it was originally described and has since been found at other nearby sites [17]. These flies live in and around bats, although they have not yet been observed as attached or riding phoretically on a bat, and they do not seem to be blood or tissue feeders. Immature stages have been recorded and reared from bat guano, and adults are found crawling around caves and fissures where bats are found [17]. *Mormotomyia hirsuta* is classified as the sole representative of the Afrotropical family Mormotomyiidae. Although *Mormotomyia* is superficially similar to the common yellow dung fly, *Scathophaga stercoraria* (L.) (Calyptratae: Scathophagidae; [14]), several authors have compared Mormotomyiidae to Sphaeroceridae and Heleomyzidae [13, 18, 19], two families that are close relatives within the acalyptrate superfamily Sphaeroceroidea [19, 20]. This relationship is perhaps suggested by the presence of similarly modified adult features found in Heleomyzidae associated with caves or birds’ nests. McAlpine & Woodley [22], however, found no convincing similarity of *Mormotomyia* with sphaerocerids and heleomyzids, and these families were combined in D.K. McAlpine’s concept of the family Heteromyzidae, although still classified separately by most workers [19, 21].As *Mormotomyia* exhibits characteristics of both calyptrate and acalyptrates [14], Hennig [22] cited instead a possible position as sister group to Calyptratae. In their more recent re-evaluation of these flies, Kirk-Spriggs *et al*. [16] noted features of the female reproductive tract consistent with inclusion in the superfamily Ephydroidea. David K. McAlpine [23] corroborated this placement based on antennal structure. These results and the availability of freshly preserved material of *Mormotomyia*, together with unexpected findings from more broadly sampled studies [20, 24], prompted a closer look at family-level relationships of Ephydroidea using phylogenomic datasets containing hundreds of genetic loci.

**Fig 2:**
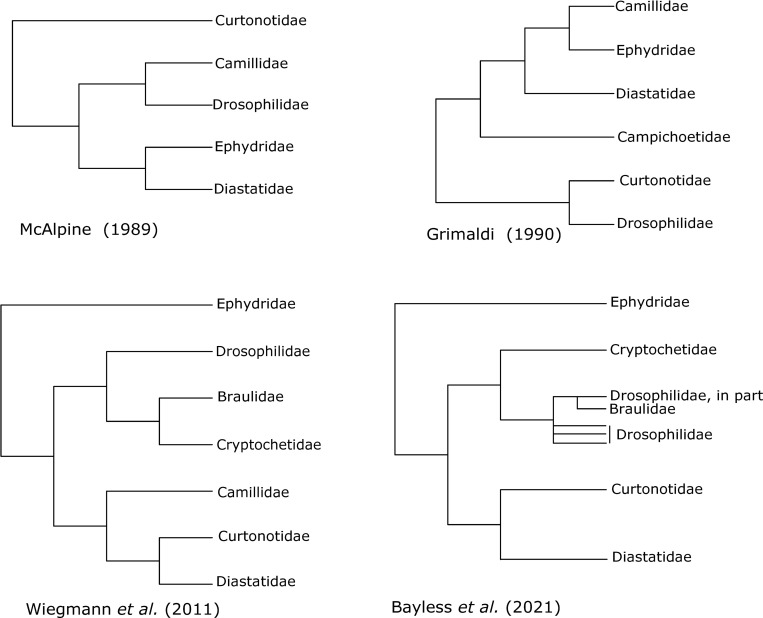
Alternative phylogenetic hypotheses for relationships among families of Ephydroidea: a) J.F. McAlpine (1989); b) Grimaldi (1990); c) Wiegmann *et al*. (2011); d) Bayless *et al*. (2021).

The superfamily Ephydroidea also includes the species-rich families Drosophilidae (vinegar flies, pomace flies and laboratory “fruit flies”) and Ephydridae (shore flies), along with the small and relatively obscure families Camillidae, Curtonotidae and Diastatidae [19] (Fig 1A–1F). The superfamily is one of only a few well-supported superfamilies of acalyptrate flies recovered in two recent molecular analyses [20, 24], which included representatives of many schizophoran fly families, but did not include Mormotomyiidae, or a more broadly representative taxon sampling within Ephydroidea. Possibly the most surprising result from recent work is the consistently strongly supported grouping of *Drosophila* Fallén together with *Cryptochetum* Rondani and *Braula* Nitzsch, the last two named representing two enigmatic families with specialized habits, the placement of which, like that of *Mormotomyia*, has been especially uncertain [19, 25] (Fig 2). Cryptochetidae are endoparasitoids of scale insects and have been successfully deployed in biological control of cushion scale insects in California and elsewhere [26, 27]. Braulidae, known as “beelice”, are closely associated with honeybees (*Apis mellifera* L. and *Apis dorsata* (Fabricius). Adults of this family have highly specialized morphological features that are classically associated with external parasitism or phoresy, including loss of eyes, reduction of antennae and wings, reduced thorax and mouthparts, comb like claws, and dense hairs and or bristles (Fig 1F). Larvae burrow through the honeycomb feeding on wax, honey and pollen, while the wingless adults cling to the body of worker bees and steal regurgitated nectar [28, 29]. Both families had been included in the superfamily Carnoidea, with filth flies (Carnidae) grass and frit flies (Chloropidae), beach flies (Canacidae) and a number of more obscure families with restricted geographic ranges [19, 30]. A relationship of Braulidae and Cryptochetidae with Drosophilidae and related families has, in fact, been previously proposed. In the case of Cryptochetidae, the evidence for this relationship comes from two obscure genera inconclusively assigned to the family, one (*Phanerochaetum* Hennig) a Baltic amber fossil (37.8–33.9 MYA; [31]) and the other (*Librella* McAlpine) known from a handful of female specimens from Australia [32]. Exhibiting a mosaic of characters found in *Cryptochetum* and those found in Ephydroidea, these genera were interpreted by D.K. McAlpine [32] as intermediate lineages linking *Cryptochetum* to the Ephydroidea (as Drosophiloidea). James F. McAlpine [19], while acknowledging the affinities of these genera to Ephydroidea, disputed any relationship with *Cryptochetum*. Furthermore, it has been noted [33], that the placement of both *Braula* and *Cryptochetum* with ephydroid families is consistent with prothoracic structure as reported by Speight [34].

Another often overlooked “orphan” fly taxon is the genus *Risa* Becker, sometimes given status as the family Risidae [13, 14]. Although the biology of *Risa* is poorly understood, Papp [35] noted rearing records from the plant *Halogeton* (Amaranthaceae), with one specimen recorded as a parasitoid reared from a caterpillar on the same plant. Originally included in Milichiidae, Papp [36] suggested family status and a close relationship with Ephydridae. This revised view was not accepted by J.F. McAlpine [19], who authoritatively asserted: “Certainly it is excluded from the Ephydroidea on the basis of the different structure of its antennae, its mouth-parts, and its frontal bristling.” Nevertheless, a position within the Ephydroidea has been suggested by several recent authors [37–40]. Freidberg *et al*. [40] further proposed that *Risa* is indeed an aberrant ephydrid, in or near the subfamily Discomyzinae, and its placement in Ephydridae has been maintained in recent classifications [41].

Contrasting with these morphologically bizarre and phylogenetically enigmatic taxa, the traditionally included ephydroid families are clearly closely related, based on multiple morphological characters [19]. Monophyly of the superfamily and its main constituent families is well supported, although with differing character interpretations in all the seminal early morphology-based classificatory treatises on Cyclorrhapha [19, 22, 24]. The morphological cohesiveness of most ephydroid clades belies a remarkable diversity of life histories and biologies. This is especially true of the Drosophilidae. Many species are generalized saprophages, probably feeding predominantly on microbes in rotting material, but a wide diversity of feeding habits has been recorded within the family, suggesting that adaptation and ecological flexibility is a key feature of their biology [42]. Other drosophilids are specialized as fungivores, frugivores and leaf-miners, or have specialized breeding habits in flowers, rotting plant tissue, or sap of flowing tree wounds [42–45]. A few species are predators or parasites of Hemiptera:: Auchenorrhyncha and Sternorrhyncha [42, 46]. A few are predators in egg masses of spiders, frogs, or dragonflies; kleptoparasites of solitary bees; or aquatic predators of blackfly and midge larvae [42]. Three distantly related island endemic drosophilid species are known to inhabit the microbe-rich nephric grooves, gills, or mouthparts of land crabs [47–49]. Biology of the families Camillidae, Curtonotidae and Diastatidae are poorly known, but representatives of all three families have been collected in or near small mammal burrows and others are known to feed on dung or guano, generally with some degree of host specificity [50–55]. *Curtonotum* has been reared from damaged locust egg pods [54, 56–58]. Larvae of Ephydridae are mostly aquatic, feeding on algae or detritus [59]. A few genera are, however, predaceous or scavengers in corpses of various invertebrates and several others are leaf miners in aquatic plants. A few ephydrid species parallel the odd habits found in some Drosophilidae as predators in egg masses of spiders or frogs and one genus feeds on haemolymph of ants [59]. One of the most notable larval habitats of any insect is that of *Diasemocera petrolei* (Coquillett), which occurs in pools of crude petroleum, where they scavenge dead insects [59, 60]. Given this mélange of feeding habits, it is evident that the specialized life histories of Braulidae, Cryptochetidae and Mormotomyiidae, although unusual, are not “out of place” in the Ephydroidea.

Past estimates of Ephydroidea phylogeny (Fig 2A–2D) have been surprisingly incongruent, especially regarding the sister groups of Ephydridae (or Ephydridae + Risidae) and Drosophilidae. James F. McAlpine [19] followed the precedents of Hennig [18] and others, in placing Diastatidae as sister to Ephydridae [37] (Fig 2A). Griffiths [25] proposed that *Diastata* Meigen should probably be included within Ephydridae and separated the remaining two diastatid genera into the separate family Campichoetidae. Diastatidae has subsequently been treated as separate from Ephydridae and acceptance for family status for Campichoetidae has been equivocal [19, 37, 38, 61, 62]. The morphological phylogeny of Grimaldi [38] agreed in recognizing a separate Campichoetidae and placed Camillidae as the sister to Ephydridae + Risidae, as was suggested earlier by Hennig [63]. Grimaldi [38] proposed Curtonotidae as sister to Drosophilidae, where previous authors placed Camillidae as the nearest relative of Drosophilidae [19], or simply placed Camillidae, Curtonotidae and Drosophilidae in a single group [37, 61] (Fig 2B). Most recently, Yassin [33] transferred two drosophilid genera, *Cladochaeta* Coquillett and *Diathoneura* Duda,to Ephydridae based on similarities in wing venation and male terminalia, although this has not been widely accepted as it would require extraordinary loss or convergence in a large number of morphological features [64].

Considering the persistent phylogenetic confusion reviewed above and the intractability of acalyptrate relationships at deeper levels, the results of Bayless *et al*. [20] and Wiegmann *et al*. [24] for Ephydroidea were surprising (Fig 2C and 2D). In contrast to previous hypotheses, both studies recovered Ephydridae as sister to the remaining families. They also reported consistently a clade containing Curtonotidae, Camillidae and Diastatidae (including *Campichoeta*) as sister to the previously mentioned grouping of Drosophilidae + Braulidae + Cryptochetidae. Both studies raise the possibility of a paraphyletic Drosophilidae, especially with respect to the position of Braulidae, but intrafamilial taxon sampling was too sparse to resolve relationships at this level. In particular, Bayless *et al*. [20] found strong support placing Braulidae as sister to sampled representatives of the drosophilid subfamily Steganinae (Fig 2D). Considering the biological similarities shared between Braulidae, Cryptochetidae and many steganines (*e*.*g*., subtribe Acletoxenina: *Acletoxenus* preying upon Sternorrhyncha [65], *Cacoxenus* associating with bees; [38]), the possibility of a position for these family-level clades within Drosophilidae must be considered. The phylogeny of *Drosophila* and related genera (many of which are nested within the genus *Drosophila*), has recently been extensively investigated through molecular phylogenetic studies with larger samples of steganine and drosophiline genera [33, 66–69]. None of these studies concerning Drosophilidae, however, have included Braulidae or Cryptochetidae; consequently, exact delimitation of the family Drosophilidae with regard to these aberrant relatives awaits a broadly sampled family-level analysis including representatives of Braulidae, Cryptochetidae and additional ephydroid outgroups.

Nextgen phylogenomic methods, especially Anchored Hybrid Enrichment (AHE) [70] and Ultraconserved Elements (UCE) [71], comparative transcriptomics [8, 20] and draft genome sequencing [72] have rapidly expanded the availability of phylogenetic data useful for resolving relationships within rapid radiations, and for investigating enigmatic relationships, such as those described above for Ephydroidea. In Diptera, the use of Anchored Hybrid Enrichment has yielded important data sets for large families [73–75]. A Diptera specific probe kit (NCSU-Wiegmann), has been shown to capture hundreds of single-copy orthologous loci and provide unprecedented resolution for phylogenetic questions at multiple levels–from superfamilies to species. These data are also combinable with transcriptome and genomic data sets that contain most of the captured loci, making the genomic resources already available for model organisms and pest species especially valuable for increasing phylogenetic coverage [76]. Consequently, to investigate relationships among the ephydroid families and evaluate evidence for the placement of the enigmatic family Mormotomyiidae and other newly recognized representatives of the superfamily, we used the NCSU-Wiegmann AHE probes to generate a large phylogenomic analysis of diverse ephydroid clades.

## Materials and methods

### Taxon sampling and data acquisition

Specimens used in this study were obtained from colleagues or collected by the co-authors and preserved in 95% ethanol for genomic DNA extraction. In most cases, specimens were extracted by homogenization of the entire body, with exoskeletons or remaining body parts retained as vouchers where possible (Table 1). Forty-three species are sampled, including 32 from Ephydroidea and a representative diversity of non-ephydroid schizophoran Cyclorrhapha as outgroups. To provide a root for Ephydroidea, a range of outgroups were chosen with due consideration to current phylogenetic uncertainty about the monophyly and relationships of some schizophoran clades [20]. The outgroup taxa include representatives of both calyptrate and acalyptrate schizophorans–which have been placed, although sometimes without strong support, as close relatives of the Ephydroidea [20, 23, 24]. A representative of the enigmatic family Nannodastiidae was also sampled, due to its occasional inclusion in Ephydridae [77]. In all, we selected 11 outgroup taxa, including representatives of the Agromyzidae (*Liriomyza sativae* Blanchard), Odiniidae (*Odinia conspicua* Sabrosky), Nannodastiidae (*Azorastia mediterranea* Papp); Tephritoidea: Platystomatidae (*Rivellia syngenesiae* (Fabricius)), Tephritidae (*Ceratitis capitata* (Wiedemann)); Calyptratae, muscoid grade: Anthomyiidae (*Delia radicum* (Linneaus)), Oestroidea: Tachinidae (*Exorista larvarum* (Linneaus)), Sarcophagidae (*Sarcophaga bullata* Parker) and Oestridae: (*Cuterebra atrox* Clark). Within the Ephydroidea, we include multiple individuals for most of the constituent families: Braulidae (2), Camillidae (2), Campichoetidae (1), Cryptochetidae (2), Curtonotidae (4), Diastatidae (2), Drosophilidae (9), Ephydridae (9) and Mormotomyiidae (monotypic).

**Table 1:** Taxa sampled for sequencing and specimen deposition information.

Family	Superfamily	Subfamily/Tribe	Taxon	Accession Number	BioSample#	Specimen LabCode	Collecting Locality
Agromyzidae		Phytomyzinae	*Liriomyza sativae*	n/a	SAMN28901500	AG1433 -AHE	California, USA
Odiniidae			*Odinia conspicua*	SRX71220804	SAMN13151330	NCSU:Odsp- transcriptome	North Carolina, USA
Carnidae			*Carnus hemapterus*	SRX7371317	SAMN13528633	HS004-RDA003 transcriptome	Spain
Carnidae			*Meoneura* sp.	n/a	SAMN28901501	MecolRG-AHE	Colorado, USA
Platystomatidae	Tephritoidea		*Rivellia syngenesiae*	n/a	SAMN28901502	I6807-AHE	Switzerland
Tephritidae	Tephritoidea	Dacinae	*Ceratitis capitata*	n/a	SAMN28901503	I6808-AHE	Kenya
Nannodastiidae			*Azorastia mediterranea*	n/a	SAMN28901504	azmed3RG-AHE	Israel
Anthomyiidae	Muscoidea		*Delia radicum*	n/a	SAMN28901505	I6805-AHE	Ottawa,Canada AgCanada
Tachinidae	Oestroidea	Esoristinae	*Exorista larvorum*	n/a	SAMN28901506	I6806-AHE	Italy lab colony
Sarcophagidae	Oestroidea	Sarcophaginae	*Sarcophaga bullata*	n/a	SAMN28901507	I6803-AHE	Maryland,USA lab colony
Oestridae	Oestroidea	Cuterebrinae	*Cuterebra atrox*	n/a	SAMN28901508	I6804-AHE	Arizona, USA
Ephydridae	Ephydroidea	Ilytheinae: Hyadinini	*Philygria* sp.	n/a	SAMN28901509	I4191-AHE	North Carolina, USA
Ephydridae	Ephydroidea	Ephydrinae: Ephydrini	*Coenia palustris*	n/a	SAMN28901510	I3850- AHE	Hungary
Ephydridae	Ephydroidea	Ephydrinae: Ephydrini	*Ephydra hians*	SRX827012	SAMN03220590	2014-Female-whole body-transcriptome	UC Berkeley, NCBI SRA_Database Sample; California
Ephydridae	Ephydroidea	Ephydrinae: Scatellini	*Scatella lacustris*	SRX798115	SAMN03223157	GCFE01_1-transcriptome	North Carolina, USA
Ephydridae	Ephydroidea	Gymnomyzinae: Gymnomyzini	*Athyroglossa* sp.	n/a	SAMN28901511	I4186—AHE	North Carolina, USA
Ephydridae	Ephydroidea	Discomyzinae	*Achaetorisa* sp. 1	n/a	SAMN28901512	I6792-AHE	Israel
Ephydridae	Ephydroidea	Discomyzinae	*Risa longirostris*	n/a	SAMN28901513	I4192-AHE	Israel
Ephydridae	Ephydroidea	Discomyzinae: Psilopini	*Clanoneurum* sp.	n/a	SAMN28901514	I4188-AHE	Israel
Ephydridae	Ephydroidea	Discomyzinae: Psilopini	*Psilopa polita*	n/a	SAMN28901515	I6800-AHE	Switzerland
Mormotomyiidae	Ephydroidea		*Mormotomyia hirsuta*	n/a	SAMN28901516	I3857-AHE	Kenya
Camillidae	Ephydroidea		*Afrocamilla stuckenbergi*	n/a	SAMN28901517	camspRG-AHE	South Africa
Camillidae	Ephydroidea		*Camilla* sp.	n/a	SAMN28901518	I3845-AHE	United Kingdom
Diastatidae	Ephydroidea	Campichoetinae	*Campichoeta punctum*	n/a	SAMN28901519	I6795-AHE	Switzerland
Diastatidae	Ephydroidea	Diastatinae	*Diastata repleta*	SRX7122099	SAMN13151292	NCSU:diast-trancriptome	North Carolina, USA
Diastatidae	Ephydroidea	Diastatinae	*Diastata fuscula*	n/a	SAMN28901520	I6799-AHE	Hungary
Curtonotidae	Ephydroidea		*Curtonotum* sp.	SRX7122098	SAMN13111546	curtcr05-transcriptome	Costa Rica
Curtonotidae	Ephydroidea		*Curtonotum pantherinum*	n/a	SAMN28901521	cupaRG-AHE	Peru
Curtonotidae	Ephydroidea		*Cyrtona sensu lato* sp.	n/a	SAMN28901522	cyriRG-AHE	South Africa
Curtonotidae	Ephydroidea		*Cyrtona* “*Parapsinota*” sp.	n/a	SAMN28901523	cyflRG-AHE	South Africa
Cryptochetidae	Ephydroidea		*Cryptochetum* sp. 04	n/a	SAMN28901524	cry04-AHE	Australia
Cryptochetidae	Ephydroidea		*Cryptochetum* sp.	n/a	SAMN28901525	I6797-AHE	California, USA
Braulidae	Ephydroidea		*Braula coeca*	SRX1044795	SAMN03753801	GDDH01_1-transcriptome	South Africa
Braulidae	Ephydroidea		*Braula coeca*	n/a	SAMN28901526	I6794-AHE	Australia
Drosophilidae	Ephydroidea	Drosophilinae: Colocasiomyini	*Chymomyza costata*	ERX986130	ERS743378	C_costata-transcriptome	Hokkaido University, NCBI SRA_Database Sample; Japan
Drosophilidae	Ephydroidea	Drosophilinae: Drosophilini	*Drosophila melanogaster*	n/a	SAMN28901527	I3863-AHE	North Carolina, USA; NCSU lab colony
Drosophilidae	Ephydroidea	Drosophilinae: Drosophilini	*Mycodrosophila* sp.	n/a	SAMN2890152	I4190-AHE	Ohio, USA
Drosophilidae	Ephydroidea	Steganinae: Steganini	*Leucophenga varia*	SRX9518250	SAMN16729689	L_varia-transcriptome	Stanford Univ. NCBI SRA Database Sample
Drosophilidae	Ephydroidea	Steganinae: Steganini	*Leucophenga maculata*	n/a	SAMN28901529	Leucma-AHE	Hungary
Drosophilidae	Ephydroidea	Steganinae: Steganini	*Leucophenga albofasciata*	n/a	SAMN28901530	LeucalRG-AHE	Australia
Drosophilidae	Ephydroidea	Steganinae: Steganini	*Stegana* sp.	SRX7122079	SAMN13151293	GIFG01-transcriptome	Costa Rica
Drosophilidae	Ephydroidea	Steganinae: Gitonini	*Phortica variegata*	SRX827026	SAMN03220594	2014-F-RNAtranscriptome	UC Berkeley, NCBI SRA_Database Sample
Drosophilidae	Ephydroidea	Steganinae: Gitonini	*Cacoxenus australicus*	n/a	SAMN28901531	cacoxRG-AHE	Australia

### Transcriptome data

This study combines newly sequenced Anchored Hybrid Enrichment exon capture data aligned with some taxa represented by transcriptomes. Transcriptome data were gathered from the literature: *Ephydra hians* Say and *Phortica variegata* Fallén from Vicoso & Bachtrog [78]; *Chymomyza costata* Zetterstedt from Poupardin *et al*. [79] and all others from Bayless *et al*. [20]. We separately analyzed two samples of the species *Braula coeca*, one sequenced as a transcriptome and one sequenced by Anchored Hybrid enrichment. Other representatives of the family Braulidae are rare or have more restricted distributions [28], so additional sampling was not feasible. We wanted to correct for potential biases or batch effects and thus did not combine the data for the two *Braula coeca* samples.

### Anchored hybrid enrichment laboratory methods

#### DNA extraction

Adult flies were stored in ethanol at -20°C. For DNA extraction these were rinsed for a few minutes in ultrapure DEPC treated distilled water and air-dried on tissue paper. Entire specimens or thoracic muscle samples were subsequently homogenised in 1.5 ml Eppendorf tubes and incubated on a thermoblock at 65°C for several minutes. Total genomic DNA was extracted using the DNeasy Blood & Tissue Kit (Qiagen, CA, USA) following the manufacturer’s instructions. Isolated DNA was quantified with a Qubit 3.0 fluorometer using dsDNA High Sensitivity Assay Kit (Life Technologies, Inc., CA, USA) following the manufacturer’s instructions. In a few samples with low DNA yield, nucleic acids concentrations were increased by whole genome amplification using the REPLI-g Mini Kit (Qiagen, CA, USA).

#### AHE Library construction and sequencing

For each sample, 7.9–110 ng/μL (47 ng/μL mean) DNA in 50 μL total volume was sheared to approximately 300 bp by sonication with a Covaris E220 Focused-ultrasonicator using Covaris microTUBES (Covaris, Inc., MA, USA). The sheared DNA was used as input for genomic DNA library preparation and indexing using the protocol of Meyer & Kircher [80], but modified to include a size-selection step after blunt-end repair using SPRIselect beads (Beckman Coulter, Inc., CA, USA; 0.9 × ratio of bead to sample volume). Each sample was then indexed and pooled together in groups of 48 samples. We enriched each 48-sample pool using the 57, 681 tiled, custom-designed probes contained in the Diptera AHE kit [75], an Agilent Custom SureSelect kit (Agilent Technologies, CA, USA) that targets 559 unique loci. The Diptera probe kit design is detailed in Young *et al*. [75] and is based on comparison and selection of conserved 150 bp gene regions found among seven diverse fly genomes and 14 transcriptomes. We sequenced the pooled libraries using two lanes of an Illumina HiSeq 2500 (Illumina, CA, USA) run (single read, 100 bp). All AHE laboratory procedures and sequencing were conducted in laboratory facilities of the North Carolina State University (NCSU), Department of Entomology and Plant Pathology (Wiegmann Lab) and the NCSU Genomic Sciences Laboratory (GSL).

#### Data management and assembly

AHE data were assembled, processed and analysed using methods described in Buenaventura *et al*. [73]. In order to do so, we demultiplexed raw reads using *cassava* 1.8.2 at the NCSU Genomic Sciences Laboratory and these were trimmed of adapters and low-quality sequences using *Trimmomatic* v.0.36 [81]. For each set of reads we included a locus-by-locus cleaning step to remove non-fly sequences and low-quality reads based on E-values reported by BLAST searched against the NCBI database. We used *Trinity* v.2.4 [80] to assemble the cleaned reads.

The same transcriptome assemblies from Bayless *et al*. [20] were used here, except that of the ephydrid *Ephydra hians*. Raw read data for that species was downloaded from SRA (SRR1738664, SRR1738666-69, SRR1738671). These were sequenced from whole bodies and heads for males and females and ovaries and testes by Vicoso & Bachtrog [78]. Read quality was checked with FastQC v. 0.11.5 [82] to assess whether further trimming was necessary. Trimmomatic v. 0.32 [81] was used to remove adapter contamination and low-quality sequences. Trinity v2.4 [83] was used to assemble the reads into contigs. Each sequencing experiment for *Ephydra hians* was assembled separately then merged with duplicate contigs removed by dedupe.sh in the bbtools package [84].

Single-copy orthologs were confirmed for loci included in phylogenetic analyses using the program *Orthograph* v.0.5.14 [85], which uses a Hidden Markov Model-based search optimization step to assign orthology of each identified sequence to known gene models. We used the reciprocal BLAST hit criterion in *Orthograph* and all other default settings to assign loci using the Diptera: Brachycera set of 6,192 single-copy nuclear gene orthologs available on the public database OrthoDB [86] based on brachyceran genomes currently uploaded therein. In order to be included in the ortholog set in OrthoDB, a locus must be single copy in at least 90% of included genomes and present in at least 90% of these genomes. As an additional cleaning step, we selected files containing very low numbers of reads to be rechecked for contamination by BLAST searching them against a custom database of microbial sequences and against the NCBI database [87]. We followed the procedure of Andrade Justi *et al*. [88] to refine the ortholog set by removing any duplicate genes and refining through broad comparison single copy ortholog gene models from the annotated genomes in seven schizophoran Brachycera: *Ceratitis capitata*, *Drosophila melanogaster* Meigen, *Glossina austeni* Newstead, *Lucilia cuprina* (Wiedemann), *Musca domestica* Linnaeus and *Stomoxys calcitrans* (Linneaus). This ortholog set we hereafter refer to as the “BrachyBase” set (available for download on dataDRYAD.org). We retained any orthologous gene set found in 70% or more of samples for further analysis. Multiple sequence alignments (MSAs) were carried out using *MAFFT* (v.7.273) with the *L-INS-I* algorithm and the *addfragments* flag [89] on FASTA files of amino acid sequences from each orthologous gene set. We followed the procedure of Pauli *et al*. [90] to assess alignment quality by using the *addfragments* algorithm in *MAFFT* to refine alignments through identification of outlier sequences and removing outliers from both amino acid MSAs and nucleotide sequences [91]. Ortholog sequences from reference species were removed from all MSAs, and empty or X-only data columns in each alignment were removed using TrimAL [92] with the backtranslate option to also generate corresponding nucleotide MSAs from the trimmed amino acid MSAs. Next, we used an automated distance-based method to remove highly divergent individual gene sequences from alignments (R scripts available on dataDRYAD). Alignments were concatenated using custom Python scripts leveraging Biopython [92] functions (available on dataDRYAD).

#### Phylogenetic analyses

Trees were reconstructed for concatenated datasets by applying Maximum Likelihood searches in IQTREE (v. 1.4.2 and 1.4.4) [93, 94] implemented on the CIPRES Science Gateway V 3.3 (phylo.org) or the NC State University High Performance Computing Cluster. Our IQTree analyses were carried out with a partition-based approach using the edge-proportional partition model to allow partitions to have evolved under different evolutionary rates (option -ssp). Each gene locus, identified in orthology search (above) and aligned was considered a separate partition for evolutionary model testing. Models were assessed for optimality in the ModelFinder program incorporated into the IQTREE program set [95, 96] and assessed for each datatype: amino acids (AA), nucleotides (NT123) and NTcoding sites (NT12). Each search was carried out with 1000 replicate ultrafast bootstrap replicates and a single branch SH-like approximate likelihood ratio test (SH-aLRT) to obtain alternative support values for each node in the tree.

ML tree search was also carried out for each locus to obtain gene tree estimates using IQ-TREE. Multi-species coalescent species tree analysis was carried out in ASTRAL-III [97] using gene trees (one tree search per gene) for both nucleotide (all positions) and amino acid alignments of each locus. Statistical support in ASTRAL-III is reported as local posterior probabilities (LPP) applied to each branch as a quadripartition of the tree.

## Results and discussion

From the assembled loci compared against single copy gene models in the Brachybase ortholog set, we recovered large numbers of orthologous loci retained after HMM orthology search in Orthograph–loci recovery ranged from 5560 (*Stegana* Meigen) to 87 (*Cacoxenus australicus* Chassagnard & Tsacas) (Table 1). Overall, for only five of 43 taxa fewer than ^2^/_3_ of the loci used were recovered. A total of 142 (<0.013% of total gene sequences) highly divergent gene sequences were removed from alignments. Our final concatenated dataset comprised 42 species and 320 loci with an average length of 497 bp. Only 17% of the matrix was comprised of missing data cells, which could indicate loci not recovered for a particular taxon or partial marker recovery. In total, our phylogenetic data alignment comprised 478, 827 base pairs for the NT123 set, and 159, 609 amino acids in the AA set.

**Fig 3:**
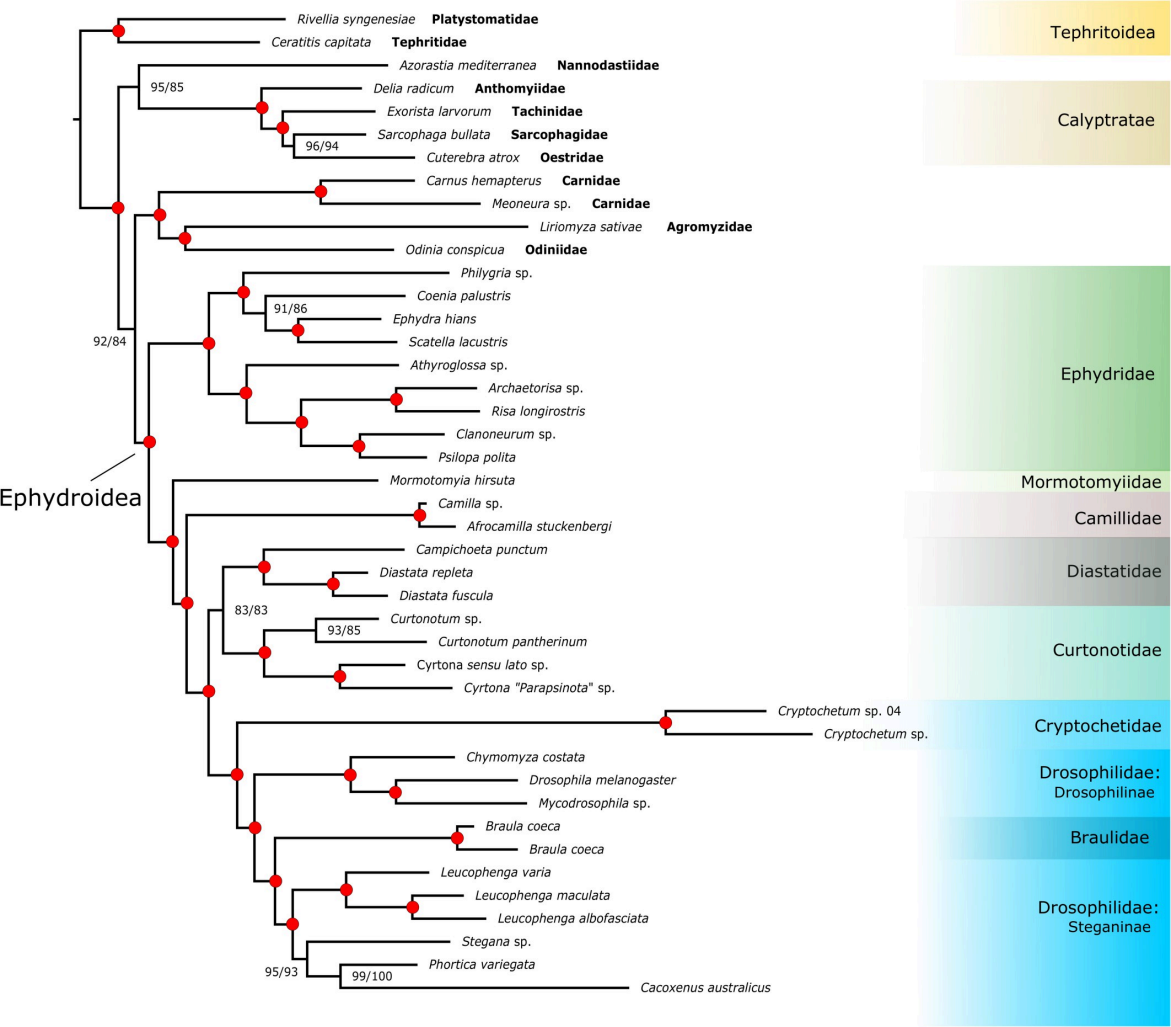
Phylogenetic tree based on concatenated amino acid data from 320 nuclear gene loci calculated in IQTREE. Branch support values are modified Shimodaira-Hasegawa Likelihood Ratio Test (SH-aLRT) support / bootstrap percentage from 1000 replicate ultrafast bootstrap searches. Red dots at nodes represent Shimodaira-Hasegawa Likelihood Ratio Test (SH-aLRT) support / bootstrap percentage of 100/100.

The Maximum Likelihood tree recovered in IQTree for the concatenated amino acid (AA) dataset is illustrated in Fig 3, with bootstrap and SH-aLRT values. Monophyly of Ephydroidea, including *Mormotomyia*, is convincingly supported, with 100% bootstrap and SH-aLRT values, as are nearly all nodes in the tree. The general pattern of relationships among families is also well-supported with Ephydridae, including the genus *Risa*, originating at the earliest split in the tree. *Mormotomyia* is placed sister to a clade consisting of all other non-ephydrid Ephydroidea. Camillidae branches next among the remaining families. Diastatidae (including *Campichoeta*) is sister to Curtonotidae and this clade is sister to a clade comprised of Braulidae, Cryptochetidae and Drosophilidae. Braulidae are strongly supported as nested within the Drosophilidae, placed as sister to Steganinae. ML analysis of the NT12 dataset in IQTree yielded a tree with identical branching patterns to that obtained for amino acids. When third position sites are included (NT123), the topology is largely congruent, except that Camillidae appears as the sister group of Braulidae, Cryptochetidae and Drosophilidae, and that the two sampled Tephritoidea genera (*Ceratitis* Macleay and *Rivellia* Robineau-Desvoidy) cluster within the Ephydroidea, disrupting the monophyly of the superfamily (S1–S3 Figs). This is likely a result of saturation of third codon positions obscuring deeper superfamily level relationships in our sample. Coalescent-based species trees calculated in Astral III (S4–S6 Figs) were also similar in topology to the well-supported tree of Fig 3, and in concatenated datasets using the same data partitions. In gene tree coalescent-based analyses relationships among schizophoran outgroup families are not strongly supported or consistently placed. Similarly low inter-family level resolution among schizophoran lineages was found by Bayless *et al*. [20], and is now a well-documented characteristic of the rapid radiation found in this part of dipteran phylogeny [24]. The Astral III analysis of individual AA gene trees yields similar results to the concatenated data result of Fig 3, except that strong support for Ephydroidea is lost, with weak support throughout the backbone of the tree. Placements among the ephydroid families are also poorly supported with increased uncertainty in the positions of Camillidae and Cryptochetidae in relation to the monophyly of the Drosophilidae.

Relationships within the families Drosophilidae and Ephydridae agree with previous studies [33, 66, 67, 98, 99], although restricted taxon sampling allows only limited tests of existing estimates of intrafamilial relationships. Our results confirm a deep divergence in Drosophilidae between Drosophilinae and Steganinae, but our placement of *Leucophenga* Mik as sister to the remaining steganines contrasts with some recent studies and needs testing by a larger phylogenomic study of relationships within the family. Finet *et al*. [67] recovered an early branching position for *Leucophenga* based on analysis of 17 mitochondrial and nuclear loci. The next step to resolve conflicting relationships and to address the limits of the richly diverse Drosophilidae is additional studies making use of broad taxon sampling, including Braulidae and Cryptochetidae and incorporating the newly available drosophilid genomic resources.

Regarding Ephydridae, our results are in general agreement with Zatwarnicki [99] in uniting Ephydrinae + Ilytheinae and Discomyzinae + Gymnomyzinae, although Hydrelliinae are not included in our sample. *Risa* and *Achaetorisa* Papp appeared as the sister to sampled species of Discomyzinae, with strong support. These results also underscore the need for additional sampling and detailed study of relationships within Ephydridae.

Although re-evaluation of broader Schizophora relationships is not the focus of this study, the position of Ephydroidea among schizophoran lineages differs between our results and those of Bayless *et al*. [20] and Wiegmann *et al*. [24]. Specifically, we did not recover a sister group relationship between Ephydroidea and Calyptratae as found in these studies with broader scope. Instead, our sampled taxon of the little-known family Nannodastiidae is placed in a grouping along with calyptrates and tephritoids, but with slightly lower bootstrap support. We attribute this difference to the limited outgroup sampling included here, noting that data sets under-sampled for either genes or taxa are highly susceptible to bias [100], and this study was designed to address relationships among putative members of a monophyletic Ephydroidea. Bayless *et al*. [20] found generally strong support across multiple analysis types and datasets in support of Ephydroidea as sister to the Calyptratae, but also showed a sizeable fraction of conflicting signal and several analysis types that supported alternative placements. Acalyptrate relationships are among the most challenging in insects and detailed studies with much more comprehensive sampling are forthcoming from a transcriptome-based study that will address higher-level Diptera relationships and divergence times using thousands of loci (Wiegmann *et al*. in prep.).

The two most significant and novel findings of our study are the firmly supported placement of *Mormotomyia* (Mormotomyiidae) within Ephydroidea, and the placement of Braulidae within the Drosophilidae. This new phylogenomic support provides strong evidence for the placement of these highly specialized and morphologically aberrant flies. This use of genomic data to help resolve challenging morphology-based conflict is similar to results for other “relict” species-poor fly lineages, such as the nematoceran families Deuterophlebiidae, Nymphomyiidae and Perissommatidae [24], the eremoneuran group Apystomyiidae [101], and the calyptrate families Mystacinobiidae and Ulurumyiidae [102]. The aberrant, specialized, highly reduced, or plesiomorphic morphology of these lineages confounded their phylogenetic placement using traditional character sets, and molecular data have recently confirmed their position as separate lineages outside of the main radiations of major clades.

A noteworthy aspect of multiple lineages within Ephydroidea are the multiple and independent origins of parasitic or highly specialized feeding habits. Similar, but convergent adaptations to a phoretic or ectoparasitic lifestyle seem apparent in Braulidae and Mormotomyidae, including reduction of compound eyes, loss or reduction of wings, and modification of legs for grasping. In fact, these attributes, as well as the characteristic “hairy*”* appearance of *Mormotomyia*, are common in many fly families among groups that are cavernicolous (cave-dwelling), inquilines of rodent or birds’ nests, or restricted to life in extreme environments, such as oceanic islands, high elevations or shorelines, as in various genera of Drosophilidae and Ephydridae [103]. Understanding how these traits are directed by the genome and shaped by selection are major biological questions, and the proximity of Cryptochetidae and Braulidae to the experimental model *Drosophila melanogaster* opens many avenues of investigation. These studies will benefit from expansion of genomic sampling outside Drosophilidae into the non-model ephydroid lineages. Comparative genomic studies are already being used within Drosophilidae to understand the interaction of ecological trade-offs, behavior and gene family evolution resulting in dietary specialization in herbivorous [104], cactophilic [105], frugivorous [106, 107] and fungivorous [108, 109] groups (see also [110]). These same methods and systems, especially when coupled with increased fieldwork aimed at more fully characterizing the biology of these groups, will allow examination of the genomics and behavior of specialization to parasitoid habits in comparisons between lineages of Drosophilidae, including Braulidae, and their (now confirmed) sister family Cryptochetidae.

## Supporting information

S1 Fig Maximum likelihood tree based on concatenated nucleotide positions 1+2. Data are from 320 aligned nuclear gene loci, partitioned by nucleotide position and gene locus for model selection in ModelFinder [94], and calculated in IQTREE. Branch support values are modified Shimodaira-Hasegawa Likelihood Ratio Test (SH-aLRT) support / bootstrap percentage from 1000 replicate ultrafast bootstrap searches.
(TIF)

S2 Fig Maximum likelihood tree based on concatenated nucleotide positions 1+2+3. Data are from 320 aligned nuclear gene loci, partitioned by nucleotide position and gene locus for model selection in ModelFinder [96], and calculated in IQTREE. Branch support values are modified Shimodaira-Hasegawa Likelihood Ratio Test (SH-aLRT) support / bootstrap percentage from 1000 replicate ultrafast bootstrap searches.
(TIF)

S3 Fig Maximum likelihood tree based on concatenated nucleotide data from 320 nuclear gene loci. The data set is partitioned only by gene locus for model selection in ModelFinder [96] and calculated in IQTREE. Branch support values are modified Shimodaira-Hasegawa Likelihood Ratio Test (SH-aLRT) support / bootstrap percentage from 1000 replicate ultrafast bootstrap searches.
(TIF)

S4 Fig Multispecies coalescent tree (MSC) from ASTRAL-III based on nucleotides 1+2. Maximum likelihood trees from each of 320 aligned loci each with a best-fitting model selected in ModelFinder [96] were calculated in IQTREE and summarized under the MSC in ASTRAL-III. Node support values are local posterior probabilities (LPP).
(TIF)

S5 Fig Multispecies coalescent tree (MSC) from ASTRAL-III based on nucleotides1+2+3. Maximum likelihood trees from each of 320 aligned loci each with a best-fitting model selected in ModelFinder [96] were calculated in IQTREE and summarized under the MSC in ASTRAL-III. Node support values are local posterior probabilities (LPP).
(TIF)

S6 Fig Multispecies coalescent tree (MSC) from ASTRAL-III based on amino acid alignments (AA). Maximum likelihood trees from each of 320 aligned loci each with a best-fitting model selected in ModelFinder [96] were calculated in IQTREE and summarized under the MSC in ASTRAL-III. Node support values are local posterior probabilities (LPP).
(TIF)
