# Supplementary figures and images for: Phylogenetic resolution of the fly superfamily Ephydroidea–Molecular systematics of the enigmatic and diverse relatives of Drosophilidae
Source: PLoS One. 2022 Oct 5;17(10):e0274292. doi: 10.1371/journal.pone.0274292 (PMC9534441; doi:10.1371/journal.pone.0274292)

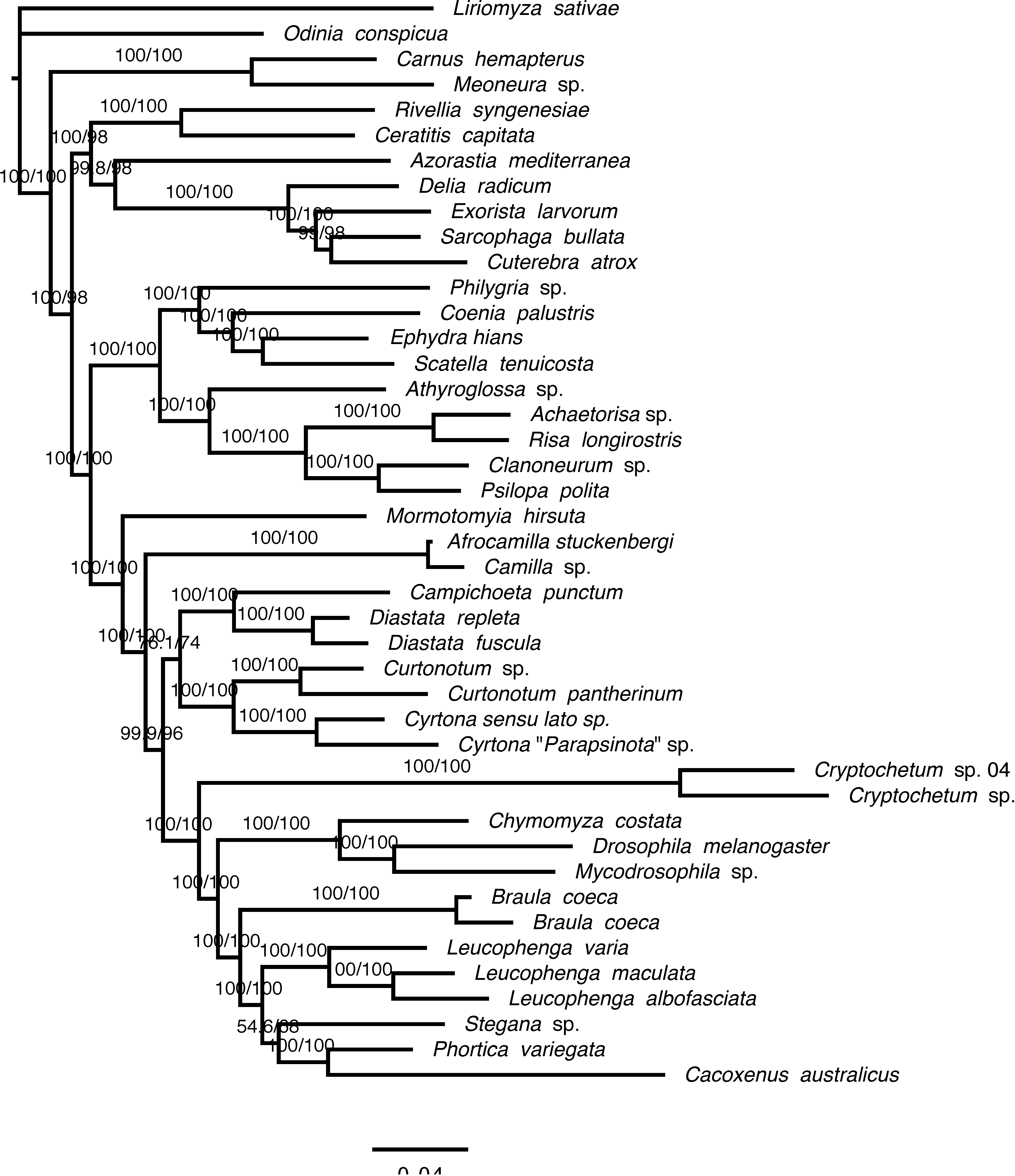

Supplement: S1 Fig — Data are from 320 aligned nuclear gene loci, partitioned by nucleotide position and gene locus for model selection in ModelFinder [94], and calculated in IQTREE. Branch support values are modified Shimodaira-Hasegawa Likelihood Ratio Test (SH-aLRT) support / bootstrap percentage from 1000 replicate ultrafast bootstrap searches. (TIF) [file pone.0274292.s001.tif]

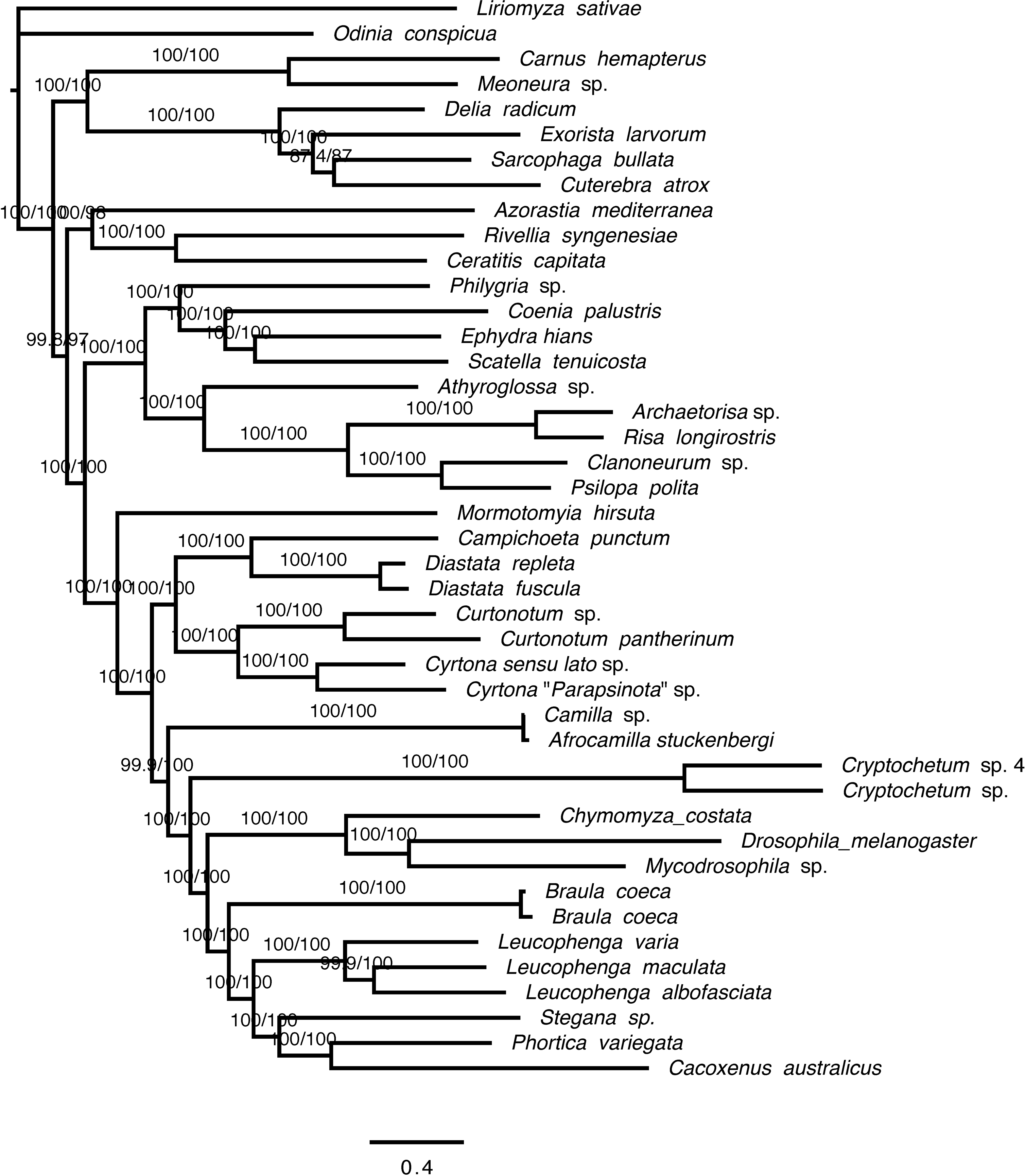

Supplement: S2 Fig — Data are from 320 aligned nuclear gene loci, partitioned by nucleotide position and gene locus for model selection in ModelFinder [96], and calculated in IQTREE. Branch support values are modified Shimodaira-Hasegawa Likelihood Ratio Test (SH-aLRT) support / bootstrap percentage from 1000 replicate ultrafast bootstrap searches. (TIF) [file pone.0274292.s002.tif]

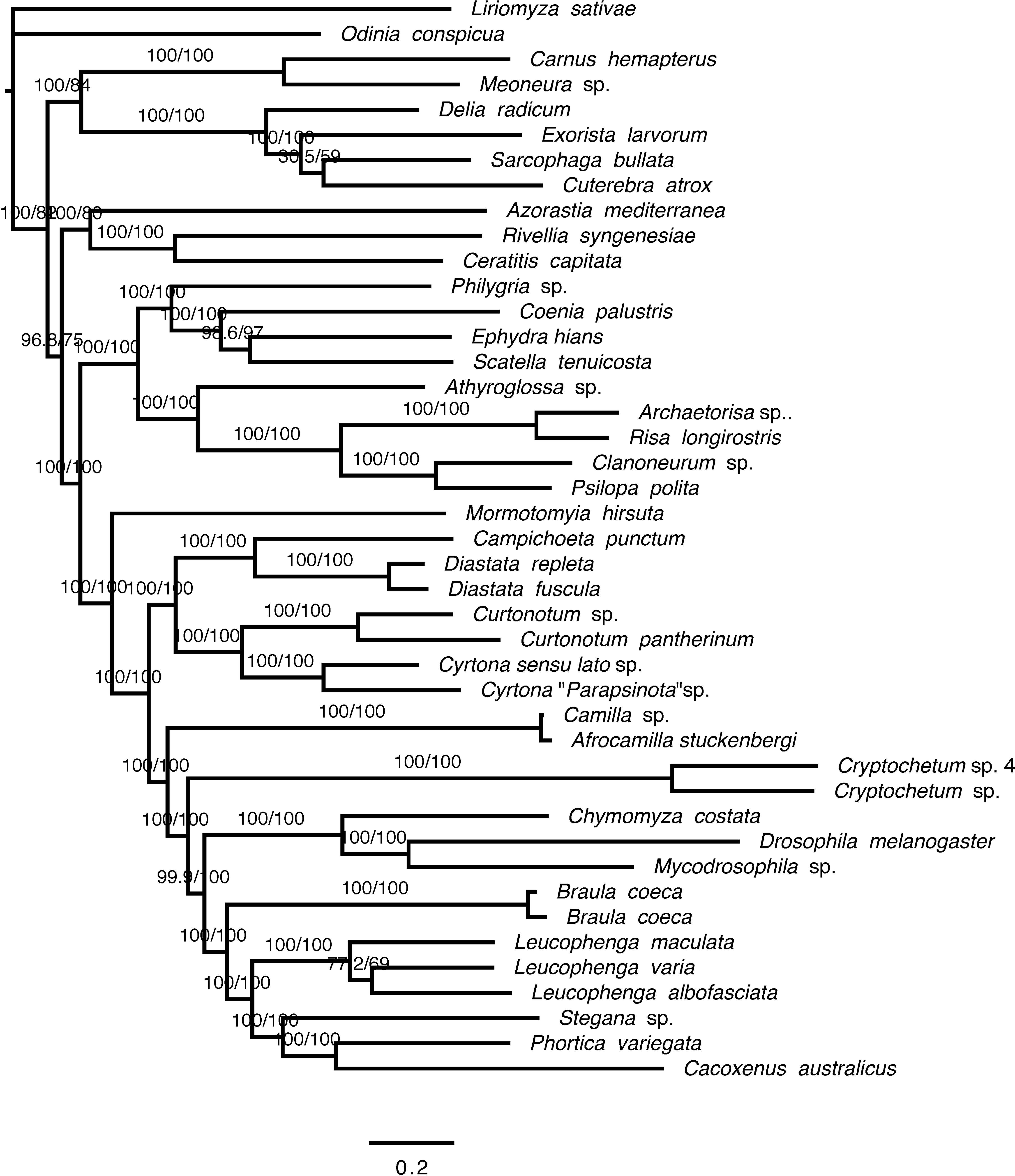

Supplement: S3 Fig — The data set is partitioned only by gene locus for model selection in ModelFinder [96] and calculated in IQTREE. Branch support values are modified Shimodaira-Hasegawa Likelihood Ratio Test (SH-aLRT) support / bootstrap percentage from 1000 replicate ultrafast bootstrap searches. (TIF) [file pone.0274292.s003.tif]

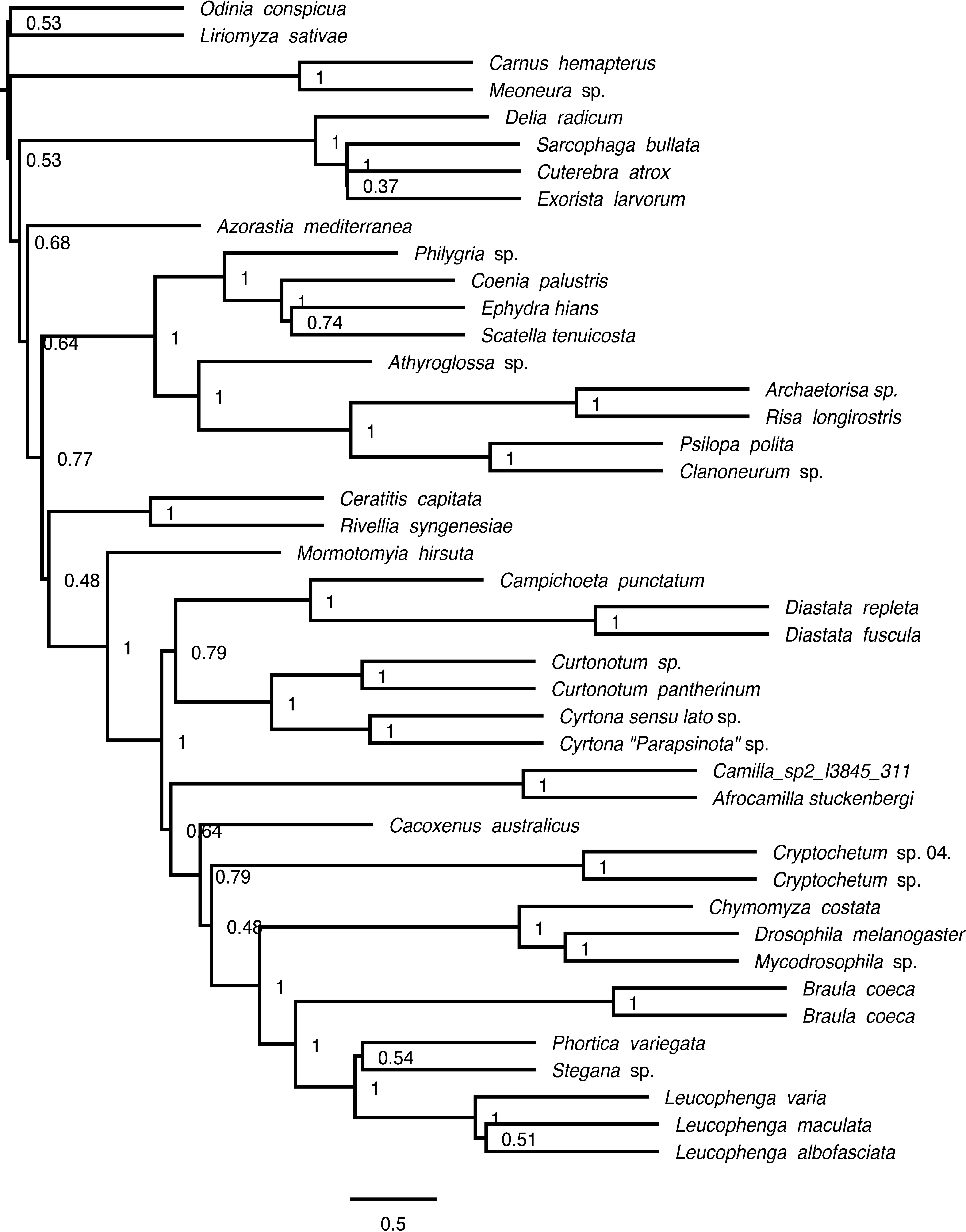

Supplement: S4 Fig — Maximum likelihood trees from each of 320 aligned loci each with a best-fitting model selected in ModelFinder [96] were calculated in IQTREE and summarized under the MSC in ASTRAL-III. Node support values are local posterior probabilities (LPP). (TIF) [file pone.0274292.s004.tif]

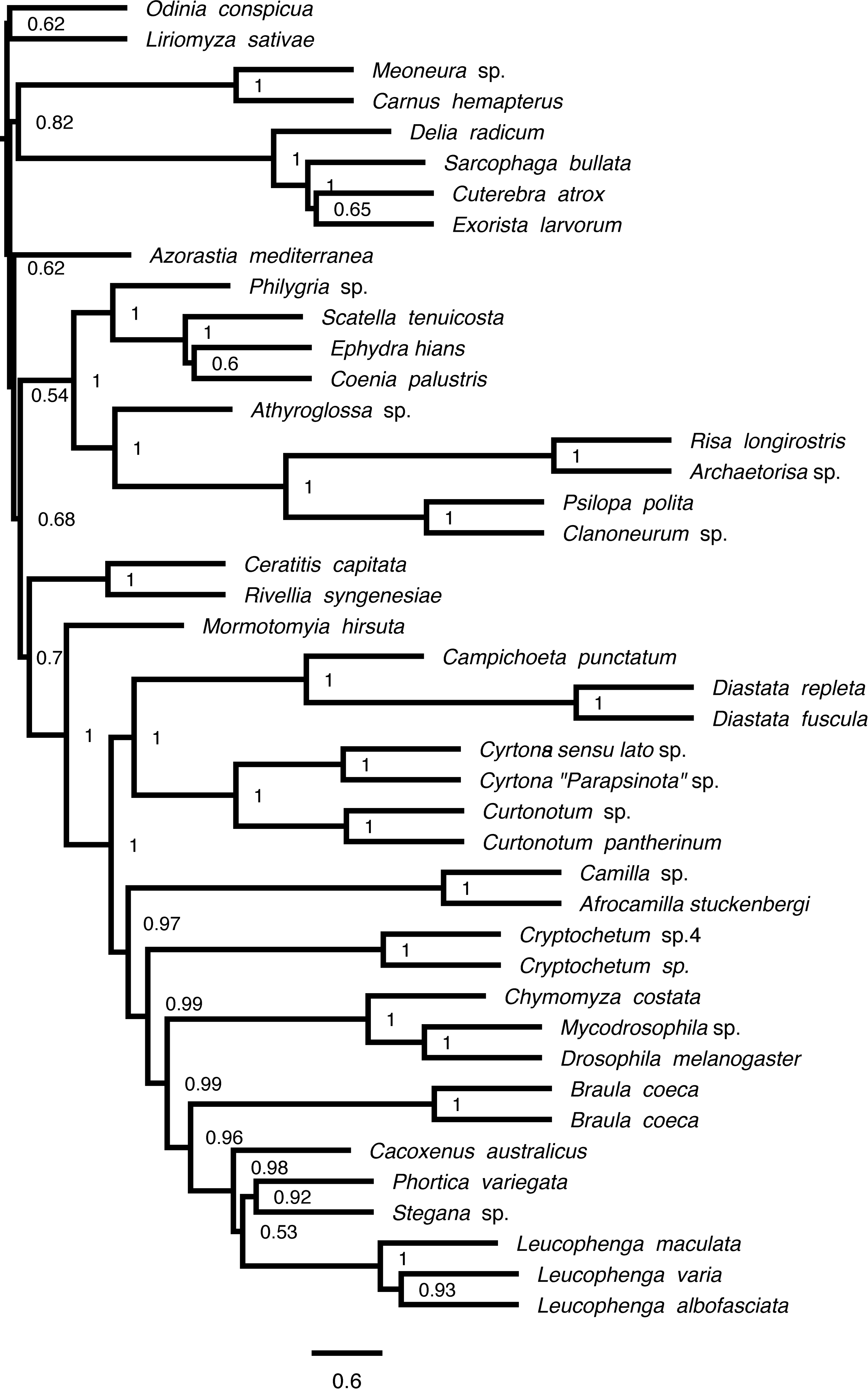

Supplement: S5 Fig — Maximum likelihood trees from each of 320 aligned loci each with a best-fitting model selected in ModelFinder [96] were calculated in IQTREE and summarized under the MSC in ASTRAL-III. Node support values are local posterior probabilities (LPP). (TIF) [file pone.0274292.s005.tif]

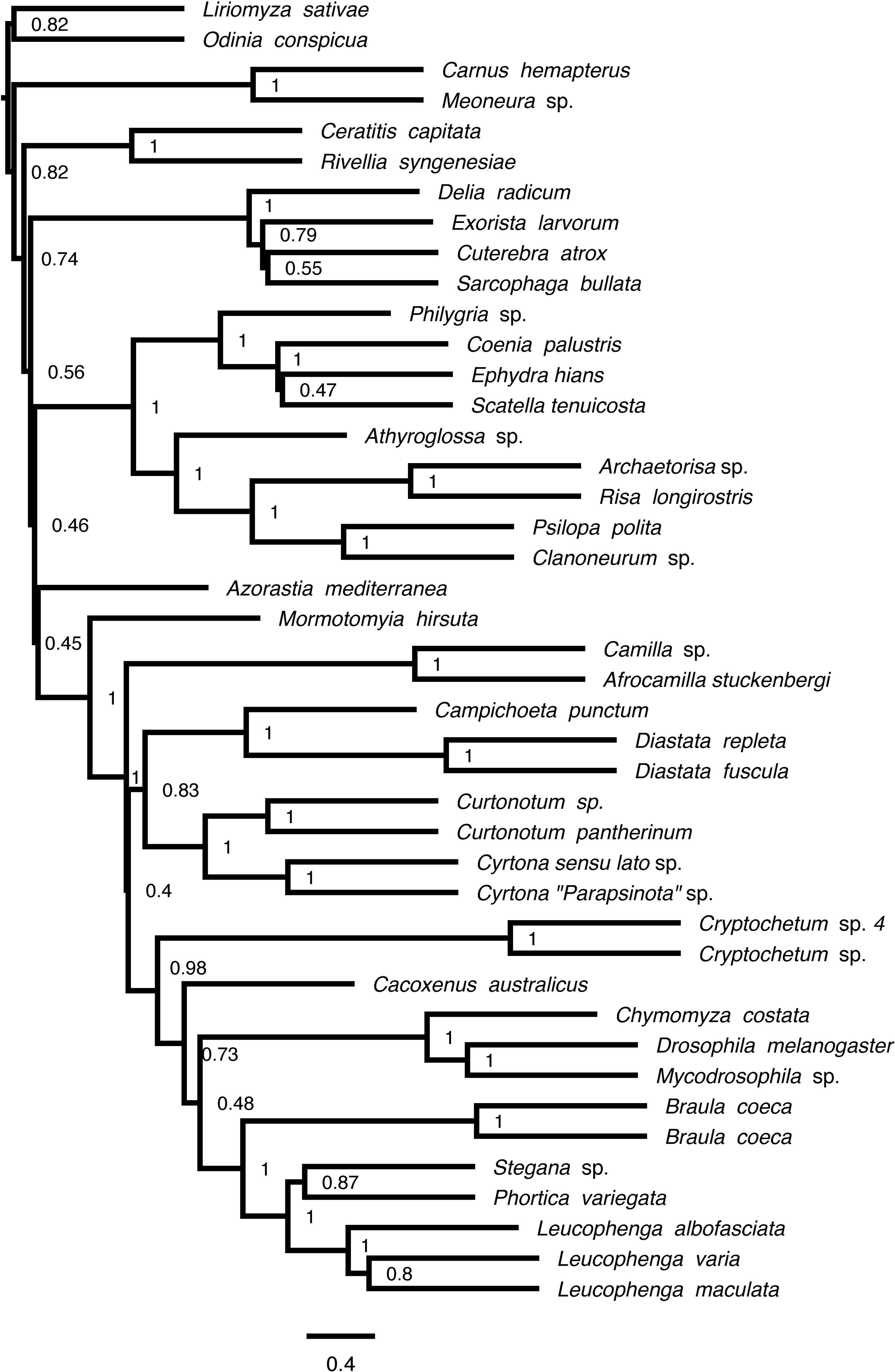

Supplement: S6 Fig — Maximum likelihood trees from each of 320 aligned loci each with a best-fitting model selected in ModelFinder [96] were calculated in IQTREE and summarized under the MSC in ASTRAL-III. Node support values are local posterior probabilities (LPP). (TIF) [file pone.0274292.s006.tif]
